# Enlarged anterior cervical diskectomy and fusion in the treatment of severe localised ossification of the posterior longitudinal ligament

**DOI:** 10.1186/s13018-016-0449-z

**Published:** 2016-10-24

**Authors:** Tao Lei, Hui Wang, Tong Tong, Qinghua Ma, Linfeng Wang, Yong Shen

**Affiliations:** Department of Spine Surgery, The Third Hospital of Hebei Medical University, The Key Laboratory of Orthopedic Biomechanics of Hebei Province, 139 Ziqiang Street, Shijiazhuang, 050051 Hebei People’s Republic of China

**Keywords:** Cervical, Anterior cervical diskectomy and fusion, Severe, Ossification of the posterior longitudinal ligament, Decompression

## Abstract

**Background:**

Severe localised ossification of the posterior longitudinal ligament (OPLL) should be directly removed by anterior approach, but the exposure during anterior cervical diskectomy and fusion (ACDF) is restricted and may increase the risk of a cerebrospinal fluid (CSF) leak. Corpectomy is facilitated to extirpate the ossification, but it is relatively more invasive. The purpose of this study was to investigate the feasibility and clinical outcome of enlarged ACDF in treating severe localised OPLL.

**Methods:**

Twenty-four selective patients with severe localised OPLL who underwent enlarged ACDF from January 2011 to July 2013 were retrospectively investigated. The Japanese Orthopaedic Association (JOA) scales, visual analogue scale (VAS), occupying rate (OR), fused segment height (FSH), sagittal segmental alignment (SSA), range of motion (ROM), and complications were investigated.

**Results:**

After a mean 34.9-month follow-up, the mean JOA score increased from 9.5 ± 1.4 preoperatively to 14.1 ± 1.5 at the final follow-up (*p* < 0.05), while OR decreased from 58.9 ± 6.1 % pre- to 10.6 ± 5.5 % postoperatively (*p* < 0.05). The average VAS was 6.1 ± 1.8 preoperatively and 2.1 ± 1.4 at the final follow-up (*p* < 0.05). The SSA angles at the final follow-up increased 2.2° compared to the preoperative values (*p* < 0.05). The mean FSH increased 2.4 mm from pre- to postoperatively, but decreased 2.7 mm from postoperatively to final follow-up. The cervical ROM was not obviously reduced at the final follow-up (*p* > 0.05) because only one level was fixed. There were three cases of cerebrospinal fluid leakage, one case of haematoma, and one case showed transient neurological deterioration.

**Conclusions:**

Enlarged ACDF is an effective procedure for treating selective patients with severe localised OPLL. Using this technique, the retrovertebral OPLL can be removed through a one-level diskectomy and a corpectomy can be avoided.

**Trial registration:**

This study has been registered with the ResearchRegistry and the unique identifying number is researchregistry1365 (K2015-022-04). It was retrospectively registered at 21 June 2016 and the first participant to the trial was at 4 January 2011.

**Electronic supplementary material:**

The online version of this article (doi:10.1186/s13018-016-0449-z) contains supplementary material, which is available to authorized users.

## Background

Ossification of the posterior longitudinal ligament (OPLL) is a common cause of cervical myelopathy in Asian countries. Localised OPLL, with ossified mass on the disk space or at the posterior margin of vertebral body [1], is an indication of anterior decompression. However, severe OPLL with occupying rate (OR) more than 50 % poses a significant challenge for spinal surgeons. The exposure of the conventional diskectomy and intervertebral fusion is restricted, which may increase the risk of cerebrospinal fluid (CSF) leak and iatrogenic cord damage [2, 3]. Some surgeons prefer anterior corpectomy and fusion (ACCF) which is facilitated to extirpate the ossification [2, 4–6]. However, the ACCF is invasive with more intraoperative blood loss and higher postoperative complications [7, 8]. More importantly, the only partly involved vertebral body and the adjacent normal intervertebral disks were unfortunately sacrificed in the ACCF. Thus, how to safely and effectively remove the severe ossification through one-level diskectomy needs to be further studied.

In this article, a surgical technique of enlarged anterior cervical diskectomy and fusion (ACDF) was performed with common surgical instruments to excise the localised mass in selective patients, and ACCF was avoided in all cases. The clinical and radiological outcomes were evaluated.

## Methods

**Fig. 1:**
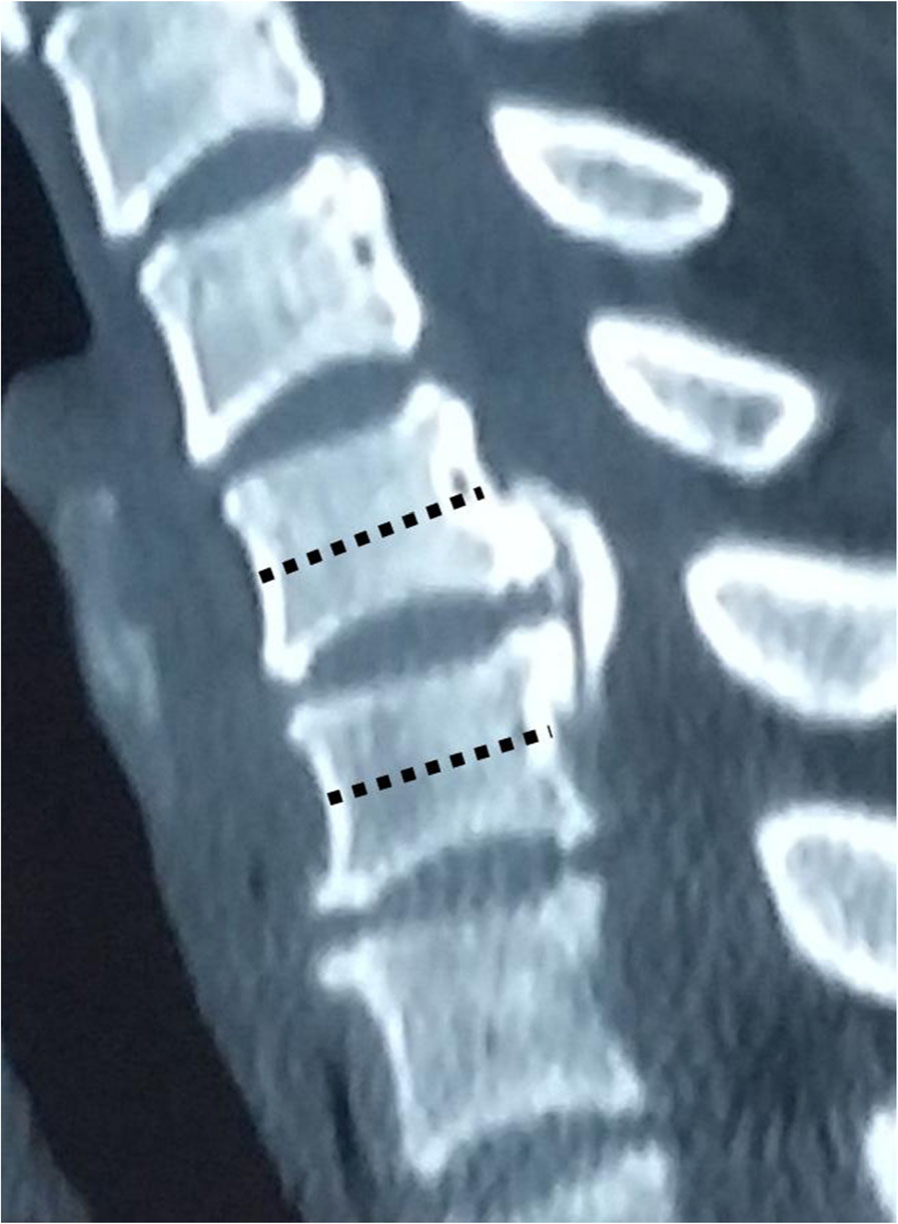
Indication for enlarged ACDF: the retrovertebral OPLL should be within half of the adjacent vertebral bodies (as *dotted line* illustrates)

**Fig. 2:**
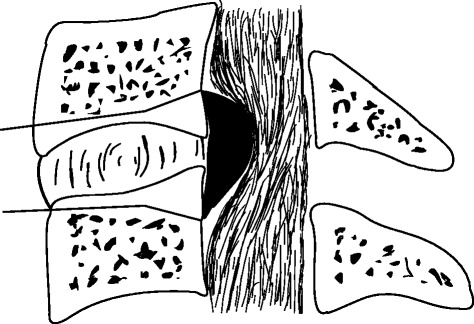
The inferior border of the cephalad and the superior border of the caudal vertebral bodies were partly excised to enlarge the intervertebral space as wedge-shaped, and the bone fragments could be used for bone grafting

Between January 2011 and July 2013, a total of 121 patients underwent surgical treatment for cervical OPLL in our department. We chose to perform anterior decompression when the pathological extent did not exceed three intervertebral levels; 24 consecutive patients presenting with localised OPLL underwent enlarged ACDF and were studied retrospectively. Inclusion criteria for this technique were as follows: (1) the retrovertebral OPLL was limited within half of the adjacent vertebral bodies (Fig. 1), and (2) the OR, defined as the maximum thickness of OPLL divided by anterior–posterior diameter of the bony spinal canal on axial CT image, was more than 50 %. Patients with myelopathy caused by ossified disk herniation or spondylosis, with cervical ossification of the ligamentum flavum, or with a history of injury or previous surgery were excluded. This study had been approved by Ethics Committee of The Third Hospital of HeBei Medical University, and all patients signed informed consent. The approval number for this study is K2015-022-04.

The study comprised 15 men and 9 women with a mean age of 54.6 ± 8.1 years (39–67 years). Mean duration of symptoms was 18.6 months. All patients had upper- or lower-limb numbness in various degrees. X-rays, CT, and MRI of the cervical spine were conducted as radiological evaluation.

### Surgical technique

**Fig. 3:**
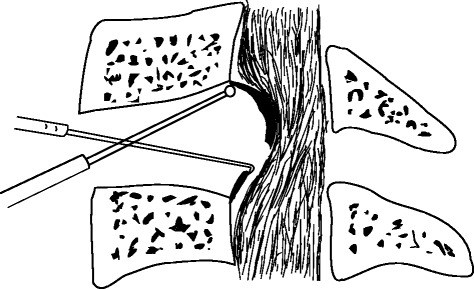
The posterior rim of the vertebral bodies was slantly polished to expose the entire OPLL and the ossified mass was slightly thinned with a burr. A microcurette was used to lever the posterior longitudinal ligament, then a gap appeared between the ossified mass and the spinal cord

**Fig. 4:**
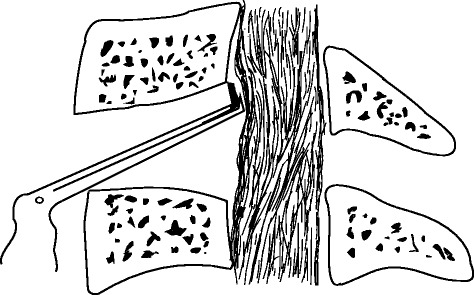
The retrovertebral ossification was excised using a 1-mm Kerrison rongeur, thus the second oppression after expansion of the dural sac could be avoided

**Fig. 5:**
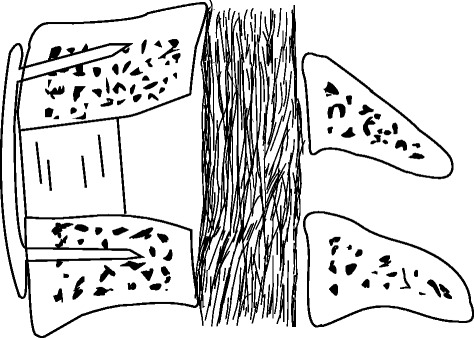
A suitable PEEK cage filled with autologous bone fragments was inserted and fixed by plate-screw osteosynthesis

Under general anaesthesia, the cervical spine was exposed through a standard right-sided approach. After confirmation via intraoperative radiography, the conventional discectomy was performed by a curette. The inferior border of the cephalad and the superior border of the caudal vertebral bodies were partly excised with an 8-mm common osteotome to enlarge the intervertebral space as wedge-shaped (Fig. 2). A drill was used to slantly polish the posterior rim of the adjacent vertebral bodies to expose the entire OPLL and to slightly thin the ossified mass (Fig. 3). A microcurette was used to lever the posterior longitudinal ligament at the lateral weak area without ossification, and a gap then appeared between the ossified mass and the spinal cord (Fig. 3). The OPLL was gently lifted and meticulously separated from the dura mater by a blunt microdissector, and then excised by a 1-mm Kerrison rongeur from the middle to either the cephalad or caudal. There was less oppression behind the posterior rim of the vertebral bodies, and the enlarged intervertebral space would facilitate undercutting the retrovertebral OPLL (Fig. 4). If the ossified mass was mainly on the right side, the operating table was leaned 10° to the left side to facilitate removing the lateral mass. In six patients, the ossified mass adhered to the dura or the dura itself was ossified; hence, the floating method was adopted. A drill was used to carefully abrade the ossified mass until it became paper thin, and the reexpanded dura mater was observed. A suitable PEEK cage, filled with autologous bone fragments harvested from excising adjacent vertebral bodies angularly, was inserted and fixed using a locking plate (Fig. 5).

### Clinical and radiological evaluation

Follow-up was conducted in all patients. Plain radiographs or CT scans were obtained at 0, 3, and 12 months postoperatively and annually thereafter. MRI scans were dependent on the clinical status.

The following parameters were investigated: (1) neurological function, evaluated by the Japanese Orthopaedic Association (JOA) scoring system; (2) neck pain, assessed with visual analogue scale (VAS); (3) sagittal segmental alignment (SSA), defined as the angle between the line along the superior endplate of the cephalad vertebrae and the line along the inferior endplate of the caudal vertebrae; (4) fused segment height (FSH), measured as the distance between the midline of the involved cranial and caudal vertebral body on radiographs; (5) range of motion (ROM) of the cervical spine was measured as the Cobb angle of C2–7 on flexion/extension lateral radiographs; (6) OR, measured on axial CT images; and (7) fusion, confirmed by the presence of trabecular bone bridging on CT scan.

### Statistical analysis

Statistical analysis was performed using SPSS version 16.0 (SPSS, Inc., Chicago, IL). Preoperative and last follow-up data were compared using paired *t* test. A *p* value less than 0.05 was considered statistically significant.

## Results

**Fig. 6:**
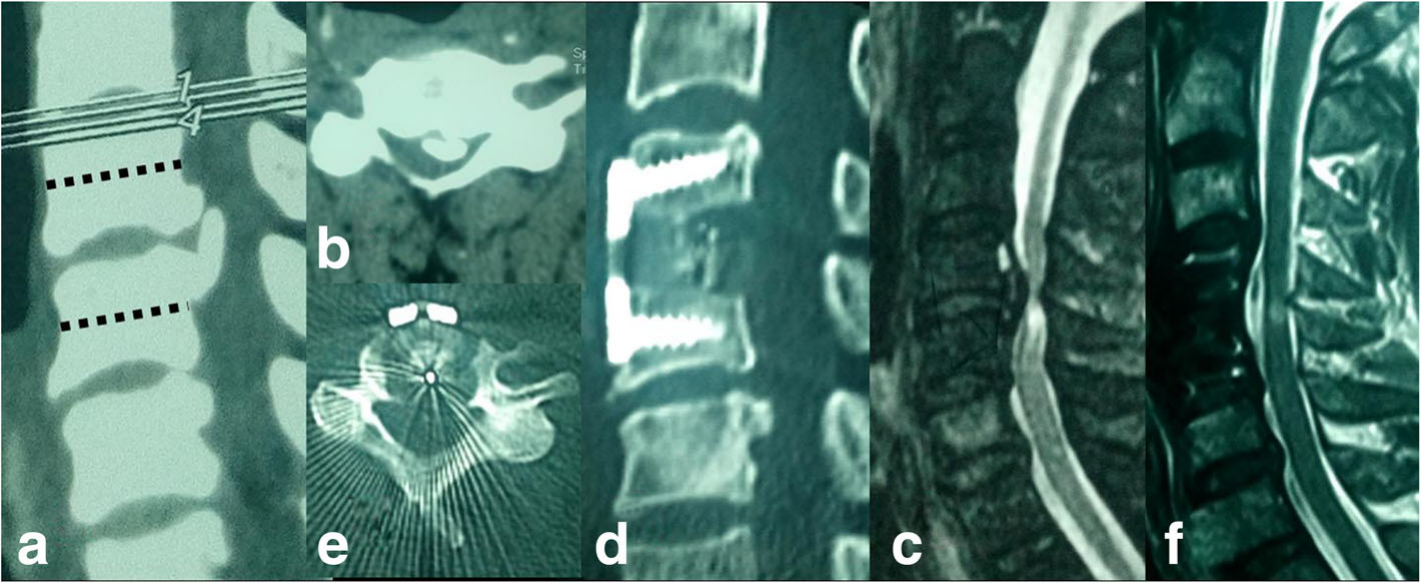
A 62-year-old man with localised OPLL received enlarged ACDF. Preoperative (**a**) lateral and (**b**) axial CT scans show OPLL behind C4/5 and part of C5 vertebral body, and the OR was 62 %. Preoperative T2-weighted (**c**) sagittal MRI show severe cord compression with increased signal intensity. Two years postoperatively, (**d**) lateral and (**e**) axial CT scans show that the intervertebral space (C4/5) was enlarged as wedge-shaped and the ossified mass was thoroughly removed. Two years postoperatively, (**f**) sagittal MRI shows adequate decompression at the C4/5 level but with residual signal intensity

Table 1 summarises the clinical data for the 24 patients. The operative levels were C4–5 in 7 patients (29 %), C5–6 in 12 patients (50 %), and C6–7 in 5 patients (21 %). The mean follow-up was 34.9 months (range, 24–52 months). The mean operative time was 108.1 ± 21.6 min, with the mean blood loss of 173.3 ± 57.1 ml. The mean JOA score increased from 9.5 (range, 7–12) at preoperation to 13.5 (range, 10–16) at the 3-month follow-up and 14.1 (range 11–17) at the final follow-up (*p* < 0.05). The average improvement rate (IR) was 64.3 % ± 15.1 %. Six (25 %) patients were graded as excellent, 14 (58.3 %) as good, and 4 (16.7 %) as fair. The average VAS was 6.1 ± 1.8 preoperatively, 3.2 ± 1.6 at the 3-month follow-up, and 2.1 ± 1.4 at the final follow-up (*p* < 0.05). The imaging of a typical case is shown in Fig. 6a–f.

**Table 1:** Clinical data before and after surgery for 24 study patients

Item	Value
Age at operative, years	54.6 ± 8.1 (39–67)
Sex	Male 15, female 9
Symptom duration, months	18.6 ± 10.3 (3–38)
Follow-up period, months	34.9 ± 7.9 (24–52)
Number of operated levels	
C4–5	7 (29 %)
C5–6	12 (50 %)
C6–7	5 (21 %)
Operative time, min	108.1 ± 21.6 (75–170)
Blood loss, ml	173.3 ± 57.1 (100–300)
JOA score	
Before surgery	9.5 ± 1.4 (7–12)
Three month after surgery	13.5 ± 1.5 (10–16)^#^
At the last follow-up	14.1 ± 1.5 (11–17)^#^
IR at the last follow-up, %	64.3 ± 15.1 (33.3–100)
Neck VAS	
Before surgery	6.1 ± 1.8 (3–9)
Three month after surgery	3.2 ± 1.6 (0–6)^#^
At the last follow-up	2.1 ± 1.4 (0–4)^#^
Complication, number of patients	
Transient minor neurological deterioration	1
CSF leakage	3
Subcutaneous hematoma	1

Values are expressed as the mean ± standard deviation (range)

^#^ *P* < 0.05, compared with the data before surgery

The radiological outcomes are shown in Table 2. The OR decreased from 58.9 ± 6.1 % preoperatively to 10.6 ± 5.5 % postoperatively, which was statistically significant (*p* < 0.001). SSA angles at the final follow-up demonstrated a slight loss of correction (1.4°) compared with postoperation, but increased 2.2° from preoperation, which was statistically significant (*p* < 0.001). The mean FSH increased 2.4 mm from pre- to postoperatively and decreased 2.7 mm from postoperatively to the final follow-up, but there was no statistical significance when compared with preoperative FSH (*p* > 0.05). Instrument subsidence, defined as a loss of FSH more than 3 mm at the last follow-up compared to postoperation, occurred in five patients (range 3.2–3.5 mm). The IR of these five patients with subsidence was 55.1 ± 13.1 % at the final follow-up, slightly less than that of the other 19 patients (66.7 ± 14.9 %), but there was no statistically significant difference (*p* > 0.05). The ROM decreased from 34.9° ± 7.7° preoperatively to 26.2° ± 7.2° at the 3-month follow-up (*p* < 0.001), but recovered to 31.4° ± 7.2° at the final follow-up without significant difference (*p* > 0.05). Solid osseous union was noted in 21 patients (87.5 %) at 3 months postoperatively. A fusion rate of 100 % was achieved at 1-year follow-up.

**Table 2:** Radiological results of surgery in 24 study patients

Item	Value
OR, %	
Before surgery	58.9 ± 6.1 (51–72)
After surgery	10.6 ± 5.5 (0–18)^#^
SSA angle, degrees	
Before surgery	0.5 ± 4.4 (−8.8–7.5)
Immediate after surgery	4.1 ± 2.9 (−0.3–8.9)^#^
Three month after surgery	3.2 ± 3.1 (−1.8–8.1)^#^
At the last follow-up	2.7 ± 3.2 (−2.4–7.9)^#^
FSH, mm	
Before surgery	32.8 ± 2.1 (29.0–37.5)
Immediate after surgery	35.2 ± 2.2 (30.9–40.0)^#^
Three month after surgery	33.4 ± 2.4 (28.4–38.0)
At the last follow-up	32.5 ± 2.4 (27.4–37.2)
ROM, degrees	
Before surgery	34.9 ± 7.7 (16.9–46.6)
Three month after surgery	26.2 ± 7.2 (11.3–39.8)^#^
At the last follow-up	31.4 ± 7.2 (16.5–45.0)

Values are expressed as the mean ± standard deviation (range)

^#^ *P* < 0.05, compared with the data before surgery

Transient minor neurological deterioration, manifesting with the weakness of right arm occurred in one case (4.2 %) after operation, but diminished after methylprednisolone pulse treatment. Intraoperatively, CSF leakage occurred in three patients due to the tight adhesion. Timely treatment was performed, including covering with artificial dura, tamping with a gelatin sponge, suturing platysma myoides densely and placing drainage beside the wound. Postoperative management included bed rest with head elevation between 10 and 20°, remove drainage until the CSF drainage volume falls below 50 ml per 24 h, continuous local pressure and anti-infection measures. The CSF leakage stopped after 3 to 5 days, and the wound healed successfully. One case (4.2 %) presented dyspnea caused by subcutaneous haematoma, which was cured by reopening the incision and later closure without neurological deterioration. There was no occurrence of instrumented failure during follow-up.

## Discussion

Ossification of the posterior longitudinal ligament (OPLL) is a common spinal disorder in Asian countries. Severe OPLL with OR more than 50 % is a great challenge for spinal surgeons because the huge ossified mass indents the spinal cord deeply. Studies have reported that the anterior approach has better outcomes and long-term benefits than the posterior approach [5, 9–12]. OPLL localised to an interspace is an indication for anterior decompression. However, ACDF is highly technically demanding because the limited operative space may increase the risk of CSF leak and iatrogenic neurological deterioration [2], and the retrovertebral OPLL below or above the disk level cannot be easily removed [3]. Though the ACCF with larger operating space was more selected to extirpate the OPLL, it was associated with longer hospital stays, greater blood loss, and more complications [2, 7, 8, 13, 14]. Another disadvantage of ACCF was sacrificing the adjacent intervertebral disk that was not involved in the localised OPLL. In this study, all patients were strictly selected on the basis of CT sagittal reconstruction with the retrovertebral OPLL less than half of adjacent vertebral bodies (Fig. 1). Enlarged diskectomy with wedge shape and polishing the posterior rim of the vertebrae provided a wide exposure of OPLL. The retrovertebral ossified mass could be removed though only one-level diskectomy, thus corpectomy could be avoided.

This procedure was similar with Williams-Isu method in the area of drilling [15, 16]. A wide operative field was yielded by resecting the vertebral bodies above and below the intervertebral space to perform decompression safely and steadily. But in our method, autologous bone graft was gained by common osteotome and placed in a PEEK cage, simplifying the complicated bone grafting in sandwich method [16]. Grauvogel [17] used piezosurgery to remove the retrovertebral osteophytes in anterior discectomy. However, the bone removal had to be done only tactually in a “blind” way [17] and the piezosurgery was not available in most institutions in China. In our technique, the polishing and resecting were performed with direct view, and the drill, curette and microdissector were instruments of daily use. The OR decreased from 58.9 % preoperatively to 10.6 % postoperatively and the IR was 64.3 ± 15.1 %. Furthermore, only one level intervertebral disk space was fixed and the cervical ROM recovered at the last follow-up through the compensation by other segments. Therefore, the conclusion could be drawn that the enlarged ACDF can achieve good functional recovery by removing the OPLL thoroughly and retaining more cervical move function.

However, controversy exists about removing the part of the adjacent vertebral body. Previous studies [18, 19] suggested that the endplate should be reserved to prevent graft subsidence. In our opinion, partly resecting the neighbouring vertebrae could provide wider operative field for decompression and autologous cancellous bone for interbody fusion. The wedge-shaped intervertebral space, with ventral narrower but dorsal wider side, could retain more centrum for reconstructing intervertebral height and sagittal segmental alignment. Although obvious subsidence was observed in five patients, the neurological recovery was not influenced. There was a slight loss of SSA angles at the final follow-up compared with postoperatively, but segmental lordosis (2.2°) was corrected from preoperatively. One reason was that intraoperative distraction with a Caspar spreader may remedy the postoperative loss. Another reason was the PKKP cage had less rigid and subsidence than titanium cage [20], and the anterior plate-screw osteosynthesis was good at obtaining the vertebral height [21], segmental lordosis [22] and initial stability. Another important reason was that graft subsidence and loss of cervical lordosis appeared to occur mainly during the first 6 weeks after surgery [23]. In the current study, solid osseous union was noted in 21 patients at 3-month follow-up, which may prevent the further loss of FSH and SSA.

With regard to the complications, there were 12.5 % patients with CSF leakage which was consistent with previous reports [24]. The main reason was the tight adhesion with dural mater or the dural ossification in severe OPLL. In anterior procedure, the first key step was to find the nonossified ligament, the weak part of OPLL [6]. If the meticulous dissection is performed from the nonossified plane, in most cases, a thin but extant dural plane will be evident and the CSF leak will be avoided. Even if the CSF leakage occurs, it could be cured by conservative treatment [5, 6, 24]. Though the neurological deterioration in one patient was minor and transient, the surgeon’s manipulation should be careful and gentle.

The following should be considered regarding enlarged ACDF. First, the selective bone cutting proportion is essential to perform safe surgery. In our experience, the retrovertebral OPLL should be within 1⁄2 of adjacent vertebral bodies. Thus, it could be removed through one-level discectomy and the residual vertebral body could be enough to be implanted with screws. Second, a short plate should be used without destroying the adjacent levels. Third, after removing of the major ossified mass, the residual osteophyte at the posterior edge of the vertebrae must be probed meticulously and removed completely. The second oppression after expansion of the dural sac could be avoided. Fourth, this technique is also effective in removing large disk herniation or extrusion, hypertrophied PLL, or any other compression anterior to the cervical spinal cord because the ossification was the most technically challenging.

### Limitation

The current study has some limitations. First, this study was only a retrospective study with a limited number of 24 patients because of the rarity of this condition. Second, long-term follow-up studies and a comparison between this method and other types of procedures are necessary, which the authors plan to conduct in the future.

## Conclusions

Enlarged ACDF is a relatively effective procedure for treating severe localised OPLL. Using this technique, the retrovertebral OPLL can be removed with common surgical instruments through one-level discectomy and the corpectomy can be avoided. The clinical and radiographic outcomes of this technique are satisfying at short-term follow-up.

## Additional file

Additional file 1: Raw Data. (XLS 31 kb)

## Abbreviations

ACCF: Anterior corpectomy and fusion
ACDF: Anterior cervical diskectomy and fusion
CSF: Cerebrospinal fluid
FSH: Fused segment height
JOA: Japanese Orthopaedic Association
OPLL: Ossification of the posterior longitudinal ligament
OR: Occupying rate
ROM: Range of motion
SSA: Sagittal segmental alignment
VAS: Visual analogue scale
